# Event History Analysis of Factors Affecting Survival of Older Adults in Taiwan

**DOI:** 10.3390/healthcare10122439

**Published:** 2022-12-02

**Authors:** Yuan-Chen Lo, Wei-Chung Hsu, Shao-Jen Weng, Yao-Te Tsai, Shih-Chia Liu, Cheng-Hsiang Lin

**Affiliations:** 1 Department of Radiation Oncology, Chung-Kang Branch, Cheng-Ching General Hospital, Taichung 40764, Taiwan; 2 Department of Industrial Engineering and Enterprise Information, Tunghai University, Taichung 40704, Taiwan; 3 Healthcare Systems Consortium, Tunghai University, Taichung 40704, Taiwan; 4 Department of International Business, Feng Chia University, Taichung 40723, Taiwan; 5 Department of Statistics, Tunghai University, Taichung 40704, Taiwan

**Keywords:** older adults, longitudinal data, survival status, risk factor

## Abstract

(1) Background: Due to rapidly increasing average age of Taiwan’s population, it is very important to analyze the factors affecting the survival of older adults. (2) Methods: In this study, the 1989 Taiwan Longitudinal Study on Aging, which lasted 22 years and consisted of seven surveys, was used. Furthermore, Cox and Aalen’s time-dependent frailty models were used to analyze factors that affect the survival of older adults. (3) Results: Based on past literature, we selected 15 important factors that were closely associated with the survival of older adults and constructed six models based on these factors. The study results showed that, in addition to background characteristics, physical and mental conditions, activities of daily living (ADL), physical performance, and self-rated health had a huge association with the survival of older adults. (4) Conclusions: We selected ten variables (age, gender, population, education level, ADL status, physical performance, self-rated health, smoking, chewing betel nut, and the presence of a spouse), and their combinations were used to generate reduced models, which could be considered as important markers that affect and predict the survival of older adults.

## 1. Introduction

The study of the rapid aging of the global population has become an important issue in the world. Related topics include the efforts to determine the longevity causes [1], elderly survival forecasting [2], the rising needs of an aging population and its fulfillment attempts [3,4,5], activities to increase lifespan through healthy aging [6], and the practical meaning of the changes in health-related behaviors [7]. Taiwan has undergone huge social changes in the last 10 years due to medical and socioeconomic improvements and has transitioned from an agrarian society to an industrial society. This social transformation was accompanied with significant changes with respect to the social environment, family, and lifestyle. The demographic structure has changed from having a high birth rate and high mortality rate 50 years ago to having a low birth rate and low mortality rate. With the dawn of the low-mortality rate era, the mean life expectancy has increased in Taiwan and more than 12% of the population in Taiwan consists of older adults (≥65 years). Taiwan was predicted to become an aged society in 2018 and is expected to become a super-aged society after 2025 [8]. An aged society and a super-aged society are ones in which the proportion of adults aged 65 or older is 14% and 20% of the entire population, respectively. In addition, the proportion of older adults with spouses will increase due to the decrease in mortality rate. However, the proportion of older adults living alone (including spouses) will increase in addition to a continuous increase in the education levels of older adults.

There are many Taiwanese and international studies on event history analysis in older adults. These studies found that the survival probability of women was higher than that of men and the survival rate of people with spouses was higher than that of those without spouses [9,10,11,12]. Liu et al. [13] used the second survey data (from 1989 to 1993) of the Taiwan Longitudinal Study on Aging to examine the effects of education level on mortality rate in older adults in Taiwan. Lin and Liu [14] also examined the survival factors for older adults and selected ten variables that predicted the survival of older adults. In recent years, Liu and Lin [15] employed the Cox proportional hazard model combined with Aalen’s time-dependent model to examine the factors affecting the survival of older adults in Taiwan. In addition, some studies [16,17] further examined survival-related factors in different cohorts of older adults in Taiwan and the success, active aging, and healthy life expectancy of the older adults.

These studies found that the risk of stroke was higher in people who were active smokers, and people who chewed betel nut in the past had a higher mortality rate due to cardiovascular diseases [18,19,20]. Ho [21] and Wang [22] examined the relationship between gender, medical behavior, and survival rate, pointing out that the risk of death was lower in women than that in men and that subjects with a longer length of hospitalization and more outpatient visits had a lower survival rate. Moreover, the presence of a spouse could reduce the risk of death in older adults [22,23]. Hsu [24] and Lan et al. [25] examined the relationships between death risk, social participation, and sleep. They found that subjects who were employed and had high social participation had better survival rates, whereas subjects with long sleep durations had a higher risk of death.

It has been shown that the disease status of older adults is associated with health status and survival rate and this could be used as one of the main measurement methods of health status in older adults [22]. Anderson et al. [26] emphasized that the probability of death did not increase with more comorbidities in older adults, and that mobility is a predictor of increased survival in older adults. Molarius et al. [27] proposed that the presence of a chronic disease is a main factor affecting functional capacity and self-rated health. Therefore, it is evident that comorbidity status and physical and mental conditions affect survival. Previous studies have pointed out that self-rated health was associated with the presence of chronic disease [22,28,29,30]. In people with diabetes, the severity of depression and disability were important factors affecting self-rated health [31]. Adams et al. [32] examined the correlation between depression and the survival of patients with cardiovascular diseases. Anderson et al. [26] pointed out that there was an extremely high correlation between cardiovascular diseases and disabilities, and that the risk of death was higher in patients with more comorbidities.

In addition, a literature review shows that previous studies on the Taiwan Longitudinal Study on Aging examined factors affecting survival in older adults [18,19,20,21,24,25,33,34], health status in older adults [35,36,37,38,39,40,41,42,43,44,45], important factors related to the development of certain diseases [28,29], and important health-related and social issues [46,47,48,49,50,51,52].

Since previous in-depth studies were based on survival analyses of the temporal data of older adults in Taiwan [14,15,16] that did not consider frailty models or time-dependent variables, this study uses Cox and Aalen’s time-dependent frailty survival models for analysis, which can accurately obtain the characteristics of factors affecting the survival of older adults in Taiwan compared with previously used models. Moreover, the Taiwan Longitudinal Study on Aging used in this study includes seven surveys (from 1989 to 2011). During the 1st to the 7th surveys conducted from 1989 onwards, the youngest subject was 82 years old, and this data is approximately close to the life span of older adults born before 1929. The survival analysis results for older adults are valuable and can provide a reference of factors that affect the survival of older adults to the relevant authorities, enabling them to respond.

## 2. Materials and Methods

Event history analysis refers to an event and the time course related to the occurrence of events, such as survival time in survival analysis and its relationship to related factors. In the last 30 years, event history analysis has been widely used in demographic, public health, and sociological studies. The “events” concerned include marriage, childbirth, divorce, disease, and occupational hazards, in addition to survival. Instead of studying “people,” we can study the “event” of a company’s bankruptcy and the “survival” of its operation length.

### 2.1. Data

In this study, the people included in the cases of the Taiwan Longitudinal Study on Aging conducted by the Taiwan Health Promotion Administration (THPA) of the Ministry of Health and Welfare constituted the study subjects. The data were technically cooperated by the former Department of Health Family Project Institute and University of Michigan Population Research Center and Institute of Elders, and referred to the questionnaire design of relevant research in the USA and Japan. The first Taiwan Longitudinal Study on Aging was conducted between April 1989 and June 1989 and the objective of this study was to construct a database of the health norms and lifestyle behaviors of older adults in Taiwan. With the exception of basic demographic variables, the data also included the health status, family and life status, financial status, leisure and entertainment, social participation, and other elderly-related variables of older adults, and an investigative study analysis that included both cross-sectional and longitudinal aspects was conducted. In this survey, equal selection probability was used to select 4412 subjects among the elderly population (aged ≥60 years) from 331 plain townships/cities and districts in Taiwan. A total of 4049 subjects completed the interview, and the interview completion rate was 91.8%. The interview and subsequent follow-up visits in 1993, 1996, 1999, 2003, 2007, and 2011 were performed by the subjects themselves, with the exception of subjects who experienced a loss of consciousness, critical illness, or deafness/mutism, in which case the family member or caregiver who understood the subject’s condition answered for them. For dead subjects, the certificate of death was collected. For subjects who did not undergo subsequent follow-ups, their identification numbers, household data, and annual death cases were used to determine their survival status in the 2011 survey. Details with regard to sampling and the questionnaire appear in the 1989 Survey of Health and Living Status of the Elderly in Taiwan: Questionnaire and Survey Design.

### 2.2. Statistical Analysis

The following analytical methods were used to perform the temporal analysis of the survival of older adults from the data on the characteristics of older adults:

1. The Cox time-dependent frailty survival model

In event history survival studies, there are shortcomings associated with the fixed proportional hazards assumed in the Cox model since the risk of death for some variables will change over time. For example, the risk of death with the presence/absence of a spouse will change with time. Therefore, in consideration of the effects of covariates on survival status, time-dependent variables were included in the model and Aalen’s nonparametric additive model was used to complement the survival model by examining the effects of temporal changes on various important factors related to the survival of older adults. The relationship between cumulative regression coefficients and time was used to examine changes in their hazard ratios. On the other hand, the Cox model was based on the assumption that the survival durations of different individuals were independent of each other. Even though this assumption may be true in many experiments, problems may arise in certain situations. For example, when studying the time of the occurrence of different nonfatal conditions in the same individual, it is extremely likely that the correlation between the survival durations of the individuals is present. Frailty is a random effect that affects every individual in the group that cannot be observed. Therefore, the frailest individual will die earlier with survivors tending to belong to “stronger” families. Frailty is a random component that was created in consideration of the differences of unobservable factors [53]. Therefore, this study used the Cox time-dependent frailty survival model to correct for the unobservable individual differences and variables with time-dependent characteristics. To elaborate, this model was used to analyze the occurrence of deaths in older adults aged ≥60 years from 1989 to 2011 and examine the factors related to the risk of death in older adults. These factors included age, education level, marital status, physical performance, and self-rated health, etc.

2. Aalen’s nonparametric additive model

Aalen [54,55] proposed the nonparametric additive model, which considers the relationship between temporal dynamic function and time correlation and uses the changes in the cumulative regression coefficient over time to describe the changes in the hazard ratio for various factors. The strength of Aalen’s model is that it provides changes in the cumulative regression coefficient for different factors. If the graph of cumulative regression coefficient is not a straight line, the variable changes with time. Therefore, we can determine the changes in cumulative regression coefficient before and after certain timepoints based on the shape of the graph. Aalen’s model can be used to examine the effects of various important factors on older adults with respect to time changes in the seven surveys conducted over 22 years in the Taiwan Longitudinal Study on Aging. The relationship plots of the cumulative regression coefficient and time can be used to examine changes in their hazard ratios.

In summary, the cases in the long-term follow-up survey data of the Taiwan Longitudinal Study on Aging conducted by the Health Promotion Administration of the Ministry of Health and Welfare constituted the subjects in this study. The results of the seven surveys conducted between 1989 and 2011 were used to examine the factors affecting the survival of older adults based on previous studies.

**Figure 1:**
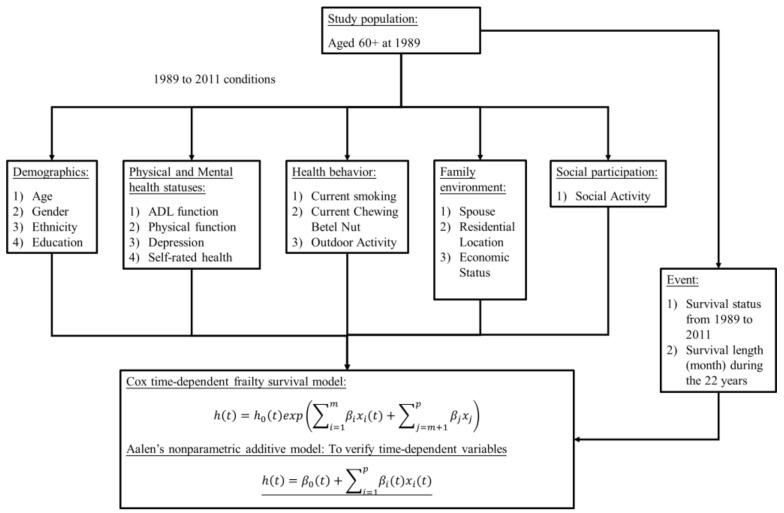
Research Framework.

We constructed a Cox proportional hazards model based on the 22-year long-term follow-up data that was supplemented by frailty and Aalen’s time-dependent frailty model for comparative analysis. The proposed research framework is shown in Figure 1. We examined 22 years of survival and death observations in more than 4000 datasets of older adults aged ≥60 years in Taiwan and analyzed the effects of independent variables such as gender, population, education level, type of residence, financial status, presence/absence of spouse, physical and mental health status, activities of daily living (ADL), depression, and type of region, etc. All analyses were carried out using SAS version 9.4 (SAS Institute, Cary, NC, USA).

## 3. Results

Table 1 shows the characteristics and statuses of older adults aged ≥60 years included in the study; of which, a two-thirds proportion of the total subjects was 60–69 years of age and one-third was aged ≥70 years. There were more males than females in a ratio of 6:4. Fukien people accounted for most of the subjects (60%), mainlanders accounted for 25%, and Hakkas accounted for 15%. There were only 1.7% of the older adults who were aborigine. Most of the older adults (80%) had an education level of up to elementary school and only 1% of the older adults had an education level of up to senior high school and above. Around 35% of the older adults did not have spouses. Nearly half of the older adults lived in cities, one-third lived in rural areas, and nearly one-fifth lived near towns. The financial status of 80% of the older adults was sufficient for monthly living expenses or more, whereas only 20% expressed difficulties in monthly living expenses. Around 75% of the older adults mentioned that their health status was good or fair, but 25% of the older adults mentioned that their health status was poor or very poor. Approximately 20% of the older adults suffered from depression. Around one-third of the total subjects were active smokers, and only 5% of the elderly subjects were active betel nut chewers. More than 90% of the older adults had participated in outdoor activities in the last 6 months, around 40% had participated in social activities, and the physical performance of most of the older adults was good.

Among the 4049 older adults interviewed in 1989, 3128 died in the subsequent 22 years, accounting for approximately 80% of the subjects (77.25%). By the time of the 2011 survey, only 921 subjects (22.75%) were still alive. It was seen that the ratio of deaths was lower in the female elderly subjects compared with that in the male elderly subjects (73.82% vs. 79.84%). A total of 1283 out of 1738 female elderly subjects and 1845 out of 2311 male elderly subjects died during the 22-year period. With regards to age group, 1789 deaths occurred in 2634 older adults aged 60–69 years, and the mortality rate was 67.92%. Among the 1415 older adults aged ≥70 years, more than 90% (1339) died during the 22-year period.

In order to understand the decrease in the number of older adults aged ≥60 years in 1989 to 2011 due to deaths, we selected 15 important factors that were closely associated with survival in older adults and constructed six Cox proportional hazard models. These models included combinations of demographic variables, physical and mental health statuses, family environment, social participation, and health behavior, and the differences between models were examined. More details of Model 1 to Model 6 are presented in Table 2. The results of these six models are shown in Table 3. We examined the relative risk of dying in older adults when various factors were present and compared the relative risk of various variables, such as the combinations of different age groups, gender, presence/absence of spouse, ADL, physical performance, and self-rated health as well as demographic variables (age group + gender + presence/absence of spouse).

With the exception of Model 1, the other five models considered the effects of physical and mental conditions, health behavior, family environment, and social participation on the survival of older adults when background characteristics were controlled. The analysis results showed that the demographic variables and education levels significantly affected survival in older adults. After adding physical and mental condition factors, the effects of depression were not found to be significant. Health behavior was significant when added as a variable. When family environment and social participation were added, only the place of residence was not significant. When all the factors were considered, the effects of depression, outdoor activities, residence, financial status, and social participation were not significant. In addition, among the background characteristics age, gender, and education level had significant effects in the six models, and it is undisputed that these variables are intimately associated with the survival of older adults. In populations, the effects of mainlanders vs. Fukien people had significant effects.

After including 15 variables that affected the survival of older adults in Taiwan, we constructed six models and compared them. However, in Model 5, factors such as depression, economic status, outdoor activities, and social participation, which are generally recognized to affect older adults, did not have significant effects. Finally, we selected the simplified model (Model 6) which included ten variables; of which, the aforementioned nonsignificant variables, such as outdoor activities, depression, economic status, residence, and social participation were eliminated. These variables were highly correlated with self-rated health, ADL, and physical performance.

The hazard description of the various variables in the simplified model (Model 6) of this study is as follows:

1. Age: The risk of death increased significantly with age and the risk of death was 4.071 times higher in subjects aged ≥70 years compared with that in those aged 60–64 years.
2. Gender: The risk of death in males was 1.912 times higher than in females.
3. Population: The risk of death in mainlanders was lower than in other populations and 0.814 times lower than that of the Fukien population.
4. Education level: The risk of death for subjects with an education level of up to senior high school and above, junior high school, and elementary school was 0.728, 0.736, and 0.826 times, respectively, compared with that of illiterate subjects. There was no significant difference between subjects with an education level of up to junior high school and those with an education level of up to senior high school. This was due to the fact that most people at that time did not have a high education level.
5. ADL status: There was no significant difference in subjects with independence and fair ADL; however, the risk of death was 2.399 times higher in subjects with poor ADL compared with those with independence in ADL.
6. Physical performance: The risk of death for subjects with common and poor physical performance was 1.578 and 2.492 times higher than that of those with good physical performance.
7. Self-rated health: The risk of death for subjects with fair and poor self-rated health was 1.207 and 1.815 times higher than that of those with good health.
8. Current smoking status: The risk of death for smokers was 1.399 times higher than that for non-smokers.
9. Current betel nut chewing status: The risk of death for betel nut chewers was 1.344 times higher than that for non-betel nut chewers.
10. Spouse: The risk of death of subjects with no spouse was 1.395 times higher than that of those with a spouse.

In addition, based on Model 6, we considered the risk of death in 12 combinations of age, gender, and spouse (controlling for effects of Ethnicity, Education, ADL, Physical Function, Self-rated Health, Current Smoking, Current Chewing Betel Nut); Table 4 shows their rankings. The risk of death in a male subject aged ≥70 years with no spouse was 10.85 times that of a female aged 60–64 years with a spouse. Hence, various variable combinations can be used to examine risk in response to different needs.

**Figure 2:**
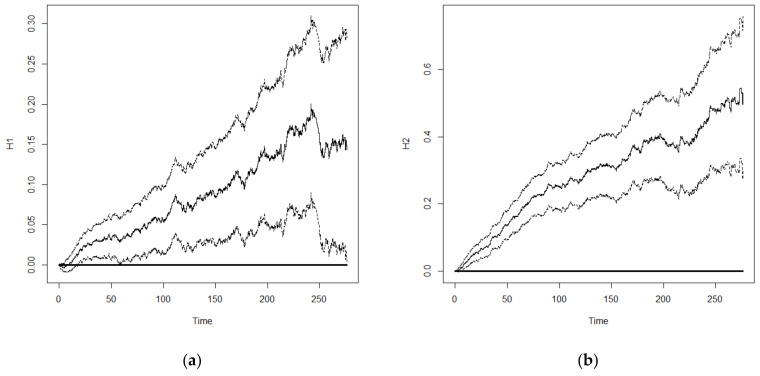
Cumulative regression coefficient and 95% confidence interval: (**a**) Fair self-rated health status; (**b**) Poor self-rated health status.

**Figure 3:**
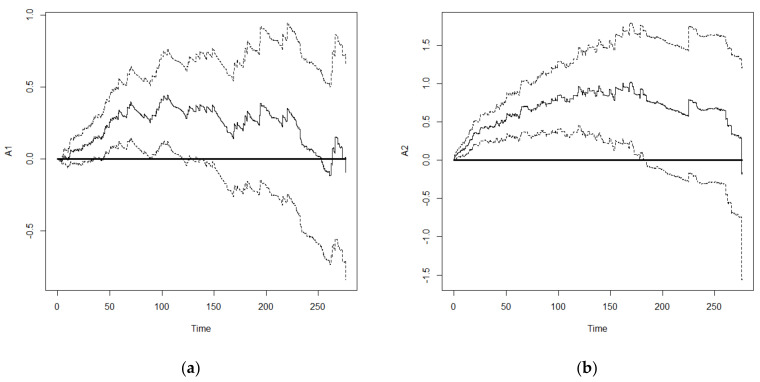
Cumulative regression coefficient and 95% confidence interval: (**a**) Slight ADL; (**b**) Poor self-rated health status.

**Figure 4:**
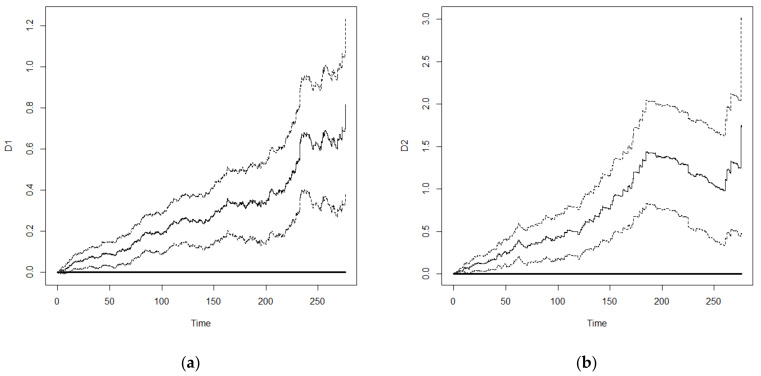
Cumulative regression coefficient and 95% confidence interval: (**a**) Common physical function; (**b**) Poor physical function.

Furthermore, we used important variables that were selected from Model 6 for comparison between the Cox frailty model and Aalen’s frailty time-dependent model. The results are shown in Table 5. Generally, the significance of important variables affecting the survival of older adults was similar between the two models. However, the analysis results from Aalen’s model could present the influence of time-dependent effects on the important variables. An important feature of the Aalen model is that its regression coefficients are allowed to vary with time. Thus, plots of the cumulative regression coefficients from Aalen’s model can be helpful in identifying the form of time-dependent effects in the proportional hazard model. The plots of the cumulative regression coefficients are expected to look different under different types of covariate effects. If a regression coefficient is constant over time, it follows that the plot of the estimated cumulative regression coefficient should look like a straight line through the origin. As self-rated health, ADL, and physical performance greatly affect survival of older adults, and the associated self-rated health is related to time, we examined the time-dependent effects of self-rated health, ADL, and physical performance. Compared with subjects with good self-rated health, the cumulative regression coefficients of subjects with fair and poor self-rated health showed a linear increasing trend with survival duration (Figure 2) but the magnitude of increase was greater in those with poor self-rated health compared with that of those with fair self-rated health. For ADL, there was no significant difference in the results between those with fair ADL and those with good ADL. Therefore, the 95% confidence interval of its cumulative regression coefficient included 0 in most survival durations (Figure 3a). On the other hand, subjects with poor ADL showed a linear increasing trend in cumulative regression coefficient before 170 months. After 170 months, subjects with poor ADL were still surviving. This might be because they received proper care and the risk of death decreased slightly, resulting in a decrease in the cumulative regression coefficient (Figure 3b). However, there was a significant difference in the risk of death between subjects with poor ADL and those with good ADL. With regards to physical performance, subjects with fair physical performance showed a linear increasing trend in the risk of death. In subjects with poor physical performance, 170 months was a threshold in which an increasing trend with occasional reduction was observed before 170 months (Figure 4), which might be similar to subjects with poor ADL. However, the decreasing trend in subjects with poor physical performance after 170 months was more significant and started to increase after 250 months. Overall, the difference in risk of death between subjects with poor physical performance and those with good physical performance was significant.

## 4. Discussion

The results of this study found that in addition to a decrease in survival probability due to older age, the survival probability of women was far greater than that of men and the survival probability of mainlanders was higher than that of other populations. This might be because mainlanders, which have a higher proportion of military, civil service, and education personnel, received better welfare. Previous studies have shown that elderly mainlanders were more receptive to institutionalized care compared with the general public [56]. On the other hand, outdoor activities, differences in the place of residence, financial status, and social activities did not result in significant differences in the survival of older adults over 22 years. With regards to health behavior, the survival probabilities of smokers and betel nut chewers were significantly lower than that of non-smokers and non-betel nut chewers.

People who smoke and chew on betel nuts are a high-risk group for contracting oral cancer. Chewing betel nuts is a unique culture in Taiwan. Betel nuts sold commercially, in addition to the areca nut, usually also contain other ingredients including betel pepper, betel leaf, betel pepper vine, slacked lime, and spices. Furthermore, guidance by the Ministry of Health and Welfare stresses that as early as 2003, the International Agency for Research on Cancer (IARC) had already proved areca nut is a group one carcinogen. In light of this, the ministry should encourage members of the public to kick the habit of chewing on betel nuts early to avoid long-term damage to their health.

There was no significant difference in the survival probability between those who participate in outdoor and social activities and those who do not. Furthermore, the simplified model used in this paper found that outdoor activities and social participation did not significantly affect the survival of older adults. This might be due to the effects of certain factors that were related to these two variables. Furthermore, social participation showed significant effects in Model 4 that did not consider physical and mental conditions. However, it was not significant in Model 5 when physical and mental conditions were added. This suggests that physical and mental conditions are closely related to outdoor activities and social participation.

With regards to physical and mental conditions, the subjectivity of self-rated health is a source of controversy over its reliability. However, there is evidence pointing out that self-rated health is a good predictor of death [57] and older adults with subjective health problems may seek medical attention. Therefore, even though self-rated health is subjective, individuals may perceive physiological signs that cannot be detected by physicians or other medical devices, and this may be the best marker for the actual condition of individuals [58,59,60]. In other words, self-rated health is mainly the ability to carry out ADL and the presence/absence, number, and severity of disease symptoms perceived by the elderly subjects, which can present the actual overall health status of an individual, and this may be a better predictor of life risk in older adults compared with objective health examination or a physician’s diagnosis. ADL can be used to assess the ability of living independently through interactions between an individual and their society and environment. Similar evaluation methods can present older adults and health-related quality of life in a more specific manner. With regards to individuals, factors such as family, the viewpoints of population service plans, and functional measurements are included as interactions with the social environment and are unaffected by age and gender. However, ADL also has its limitations as it is unable to factor in unapparent hidden conditions such as pain, emotions, satisfaction, or joy. In addition, a study pointed out that although male elderly subjects have a higher risk of death, female elderly subjects have a poor survival quality of life and the proportion of female elderly subjects with a healthy remaining life was lower than that of male elderly subjects [61]. Therefore, there still remains a need for increasing the health-related quality of life in older adults. This is necessary to complement self-rated health measurements. Evidently, the effects of gender are higher than the presence/absence of a spouse. Males aged ≥70 years are the population with the highest risk, regardless of the presence or absence of a spouse, followed by female subjects aged ≥70 years without a spouse. Hence, children have important responsibilities in ensuring a good quality of life in old age for older adults, particularly in the care of older adults of advanced age.

Two unique contributions were made. In this study, we found that the effect of depression was not significant, which did not support the previous findings [32]. The results are worth further investigation. The second contribution is that chewing betel nuts can only be observed in a few countries. Therefore, it is critical to provide strong evidence that chewing betel nuts would affect the survival of older adults. The limitation of this study is that chronic diseases were not considered.

By comparing the six constructed models, we can see which variables have important effects on the survival of older adults. Hence, the simplified model consisting of ten extremely important factors does not differ greatly compared with a full model, and only certain nonsignificant variables in the full model were eliminated. In this study, the six models can be viewed as combinations of different factors to explain the survival of older adults and a comparative analysis between the models can be performed according to different objectives.

## 5. Conclusions

This study uses the Cox and Aalen’s time-dependent frailty survival models for analysis, which can accurately obtain the characteristics of factors affecting the survival of older adults in Taiwan compared with previously used models. Moreover, the Taiwan Longitudinal Study on Aging used in this study includes seven surveys conducted between 1989 and 2011. During the 1st to the 7th surveys conducted from 1989 onwards, the youngest subject was 82 years old. This data is approximately close to the life span of older adults born before 1929. The survival analysis results for older adults are valuable and can provide a reference on factors that affect the survival of older adults for the relevant authorities and enable their response. As factors affecting the survival of older adults are relatively complex, the 15 variables of the five factors selected in this paper cannot fully present the whole picture. However, there are important markers among variables that affect the survival of older adults. The results of this study showed that the 22-year survival probability was higher in subjects with better physical performance or ADL status and similar results were seen in subjects with good self-rated health. Therefore, it can be said that the physical performance described by subjects and self-rated health are the predictors of survival after 22 years, which is consistent with the results of the ADL status. These three factors can complement each other to describe the survival status of older adults. In addition, the loss of a spouse has a considerable impact on survival in male and female elderly subjects. From the risk ranking of demographic variable combinations, it can be seen that the risk of death in female subjects without a spouse is lower than that in male subjects with a spouse in all age groups.

## Figures and Tables

**Table 1:** Characteristics and status of respondents in 1989.

Variable		*n*	%	Variable		*n*	%
Age	60–64	1482	36.60	ADL ^a^	Independence	3801	94.04
	65–69	1152	28.45	Function	Slight	93	2.30
	70–74	725	17.91		Severe	148	3.66
	75–79	438	10.82	Physical ^b^	Good	3307	81.74
	80+	252	6.22	Function	Common	510	12.61
Gender	Male	1738	42.92		Poor	229	5.66
	Female	2311	57.08	Self-rated	Good	1526	37.99
Ethnicity	Fukien	2451	60.92	Health	Fair	1491	37.12
	Hakka	603	14.99		Poor	1000	24.89
	Mainlander	900	22.37	Residential	City	1917	47.35
	Aborigine	69	1.72	Location	Town	726	17.93
Education	Illiterate	1676	41.58		Countryside	1406	34.72
	Elementary	1595	39.57	Economic	Good	1683	43.20
	Junior High	327	8.11	Status	Fair	1524	39.12
	Senior High+	433	10.74		Poor	689	17.68
Spouse	No	1429	35.31	Depression ^c^	No	3036	77.91
	Yes	2618	64.69		Yes	861	22.09
Current Smoking	No	2649	65.44	Outdoor	No	143	3.53
	Yes	1399	34.56	Activity	Yes	3906	96.47
Current Chewing	No	3825	94.58	Social	No	2485	61.37
Betel Nut	Yes	219	5.42	Activity	Yes	1564	38.63

Note: ^a^ ADL = activities of daily living, ADLs referred to the difficulty of a case in taking a bath, putting on/taking off clothes, eating, standing up from the bed or sitting on a chair, walking indoors, and going to the toilet. The lower difficulty revealed the better the ADL; ^b^ Physical functions referred to a case being able to walk to the second or third floor, walk 200–300 m, do heavy work around the house, lift something weighting 20 Taiwanese kilograms, crouch, raise hands above the head, and take or turn something with fingers. The higher value revealed better physical functions; ^c^ Depression was divided according to depression scale (CES-D), including bad appetite, bad mood, not doing well, not sleeping well, feeling happy, feeling lonely, regarding people being unfriendly, feelings of enjoying life, feeling sad, and low spirit. They were scored in 0–30, and the higher score, above, or equal to 10, was classified into depression conditions; otherwise, they were classified as not being depressed.

**Table 2:** The descriptions of Model 1 to Model 6.

Model	Description
Model 1	Demographic (Age group, Gender, Ethnicity, Education)
Model 2	Model 1 + Physical and Mental health statuses (ADL, Physical Function, Self-rated Health, Depression)
Model 3	Model 1+ Health behavior (Current Smoking, Current Chewing Betel Nut, Outdoor Activity)
Model 4	Model 1 + Family environment + Social participation (Spouse, Residential Location, Economic Status, Social Activity)
Model 5	Full Model: Demographic + Physical and Mental health statuses + Family environment + Social participation + Health behavior
Model 6	Reduced Model: Age group + Gender + Ethnicity+ Education +ADL+ Physical Function + Self-rated Health + Current Smoking, Current Chewing Betel Nut+ Spouse

**Table 3:** Results of Cox proportional frailty hazard model.

		Model 1	Model 2	Model 3	Model 4	Model 5	Model 6
Variable		HR	HR	HR	HR	HR	HR
Age	60–64	1	1	1	1	1	1
	65–69	1.632 ***	1.694 ***	1.662 ***	1.627 ***	1.682 ***	1.721 ***
	70+	3.935 ***	3.951 ***	4.162 ***	3.688 ***	3.935 ***	4.071 ***
Gender	Male	1	1	1	1	1	1
	Female	1.714 ***	2.085 ***	1.542 ***	1.874 ***	1.919 ***	1.912 ***
Ethnicity	Fukien	1	1	1	1	1	1
	Hakka	1.001	1.007	1.027	1.008	1.031	1.027
	Mainlander	0.856 **	0.828 **	0.882 *	0.821 ***	0.813 **	0.814 **
	Aborigine	1.355 *	1.287	1.149	1.384 *	1.115	1.113
Education	Illiterate	1	1	1	1	1	1
	Elementary	0.806 ***	0.852 **	0.799 ***	0.850 **	0.847 **	0.826 ***
	Junior High	0.640 ***	0.712 ***	0.649 ***	0.712 ***	0.760 **	0.736 **
	Senior High+	0.604 ***	0.658 ***	0.636 ***	0.666 ***	0.728 ***	0.728 ***
ADL	Independence		1			1	1
Function	Slight		1.326			1.266	1.318
	Severe		1.677 **			1.749 **	2.399 ***
Physical	Good		1			1	1
Function	Common		1.564 ***			1.565 ***	1.578 ***
	Poor		2.404 ***			2.445 ***	2.492 ***
Depression	No		1			1	
	Yes		1.102			1.077	
Self-rated	Good		1			1	1
Health	Fair		1.186 ***			1.189 **	1.207 ***
	Poor		1.632 ***			1.659 ***	1.815 ***
Current Smoking	No			1		1	1
	Yes			1.275 ***		1.374 ***	1.399 ***
Current Chewing	No			1		1	1
Betel Nut	Yes			1.394 ***		1.335 **	1.344 **
Outdoor	No			1		1	
Activity	Yes			2.404 ***		0.969	
Spouse	No				1	1	1
	Yes				1.297 ***	1.338 ***	1.395 ***
Residential	City				1	1	
Location	Town				1.004	0.964	
	Countryside				1.029	0.978	
Economic	Good				1	1	
Status	Fair				1.105 *	1.022	
	Poor				1.310 ***	0.992	
Social	No				1	1	
Activity	Yes				1.122 **	1.089	

Note: * *p* < 0.05; ** *p* < 0.01; *** *p* < 0.001; ADL = activities of daily living; HR = hazard ratio.

**Table 4:** The hazard ratios by age, gender, and spouse.

Rank	Age	Gender	Spouse	HR ^a^
1	60~64	Female	Yes	1.000
2	60~64	Female	No	1.395
3	65~69	Female	Yes	1.721
4	60~64	Male	Yes	1.912
5	65~69	Female	No	2.401
6	60~64	Male	No	2.667
7	65~69	Male	Yes	3.290
8	70~	Female	Yes	4.071
9	65~69	Male	No	4.591
10	70~	Female	No	5.680
11	70~	Male	Yes	7.783
12	70~	Male	No	10.85

Note: HR = Hazard Ratio; ^a^ controlled for effects of Ethnicity, Education, ADL, Physical Function, Self-rated Health, Current Smoking, Current Chewing Betel Nut.

**Table 5:** Results of Model 6 by the Cox frailty model and Aalen’s frailty time-dependent model.

		Cox Model			Aalen Model	
Variable		Coeff	SE	*p*-Value	|Z|	*p*-Value
Age	60–64 (ref)					
	65–69	0.543	0.058	<0.001	8.590	<0.001
	70+	1.404	0.060	<0.001	20.300	<0.001
Gender	Male	0.648	0.062	<0.001	9.790	<0.001
	Female (ref)					
Ethnicity	Fukien (ref)					
	Hakka	0.027	0.064	0.668	0.403	0.687
	Mainlander	-0.206	0.064	0.001	3.580	<0.001
	Aborigine	0.107	0.178	0.546	0.228	0.820
Education	Illiterate (ref)					
	Elementary	-0.191	0.056	<0.001	2.990	0.003
	Junior High	-0.307	0.096	0.001	3.130	0.002
	Senior High+	-0.318	0.092	<0.001	3.590	<0.001
ADL	Independence (ref)					
Function	Slight	0.276	0.154	0.072	1.810	0.070
	Severe	0.875	0.163	<0.001	4.200	<0.001
Physical	Good (ref)					
Function	Common	0.456	0.074	<0.001	5.130	<0.001
	Poor	0.913	0.132	<0.001	4.920	<0.001
Self-rated	Good (ref)					
Health	Fair	0.188	0.053	<0.001	3.220	0.001
	Poor	0.596	0.065	<0.001	7.280	<0.001
Current Smoking	No (ref)					
	Yes	0.336	0.056	<0.001	5.820	<0.001
Current Chewing	No (ref)					
Betel Nut	Yes	0.296	0.102	0.004	2.630	0.009
Spouse	No					
	Yes (ref)	0.333	0.050	<0.001	5.130	<0.001

## Data Availability

The data that support the findings of this study are available from the Health and Welfare Data Science Center, Ministry of Health and Welfare (HWDC, MOHW), Taiwan but restrictions apply to the availability of these data, which were used under license for the current study, and so are not publicly available.
